# Enhancement of Y_5−x_Pr_x_Sb_3−y_M_y_ (M = Sn, Pb) Electrodes for Lithium- and Sodium-Ion Batteries by Structure Disordering and CNTs Additives

**DOI:** 10.3390/ma14154331

**Published:** 2021-08-03

**Authors:** Volodymyr Pavlyuk, Wojciech Ciesielski, Nazar Pavlyuk, Damian Kulawik, Agnieszka Balińska, Karolina Kluziak

**Affiliations:** 1 Institute of Chemistry, Faculty of Science and Technology, Częstochowa Jan Długosz University, al. Armii Krajowej 13/15, 42200 Częstochowa, Poland; wc@ujd.edu.pl (W.C.); nazar.pavlyuk@gmail.com (N.P.); d.kulawik@ujd.edu.pl (D.K.); a.balinska@ujd.edu.pl (A.B.); karolina_kluziak@o2.pl (K.K.); 2 Department of Inorganic Chemistry, Ivan Franko Lviv National University, Kyryla i Mefodia 6, 79005 Lviv, Ukraine

**Keywords:** alloys, batteries, electrochemistry of materials, solid state electrochemistry

## Abstract

The maximally disordered (MD) phases with the general formula Y_5−x_Pr_x_Sb_3−y_M_y_ (M = Sn, Pb) are formed with partial substitution of Y by Pr and Sb by Sn or Pb in the binary Y_5_Sb_3_ compound. During the electrochemical lithiation and sodiation, the formation of Y_5-x_Pr_x_Sb_3-y_M_y_Li_z_ and Y_5−x_Pr_x_Sb_3−y_M_y_Na_z_ maximally disordered–high entropy intermetallic phases (MD-HEIP), as the result of insertion of Li/Na into octahedral voids, were observed. Carbon nanotubes (CNT) are an effective additive to improve the cycle stability of the Y_5−x_Pr_x_Sb_3−y_M_y_ (M = Sn, Pb) anodes for lithium-ion (LIBs) and sodium-ion batteries (SIBs). Modification of Y_5−x_Pr_x_Sb_3−y_Sn_y_ alloys by carbon nanotubes allowed us to significantly increase the discharge capacity of both types of batteries, which reaches 280 mAh · g^−1^ (for LIBs) and 160 mAh · g^−1^ (for SIBs), respectively. For Y_5−x_PrxSb_3−y_Pb_y_ alloys in which antimony is replaced by lead, these capacities are slightly smaller and are 270 mAh · g^−1^ (for LIBs) and 155 mAh · g^−1^ (for SIBs), respectively. Results show that structure disordering and CNT additives could increase the electrode capacities up to 30% for LIBs and up to 25% for SIBs.

## 1. Introduction

Intermetallics with sufficiently large interatomic voids are favorable for the insertion of lithium or sodium ions. The intermetallic compound LaSn_3_ (AuCu_3_-type) as a possible negative electrode for the lithium-ion batteries was described by Vaughey et al. [1]. The insertion of lithium atoms into octahedral voids of hexagonal Zr_5_Sn_3_ and RE_5_M_3_ (RE = Y, La and Gd; M = Ge and Sn) binary phases were described earlier by us [2,3,4]. The crystal structure of RE_5_M_3_ intermetallic compounds belongs to Mn_5_Si_3_-type. The RE atoms are located at 4*d* and 6*g* sites, respectively. The M components (Ge or Sn or Pb atoms) are located at the additional 6*g* site. For this binary structure, the presence of an octahedral void in the 2*b* site is typical. The incorporation of the third element (alkaline or transitions metals, marked as T) into the 2*b* site leads to the realization of the RE_5_TM_3_ ternary phase with a Hf_5_CuSn_3_ structure type [5]. We detected the new ternary phase of Tb_5_LiSn_3_ with fully filled 2*b* site (Hf_5_CuSn_3_ structure type) during the systematic study of the alloys of Tb—Li—Sn system [6]. The La_5_Li_x_Ge_3_ phase, which formed by partial incorporation (up to x = 0.4) of lithium atoms into octahedral voids of the La_5_Ge_3_ binary, was observed in La—Li—Ge system [3].

The rationale for the use of high entropy alloys as electrode materials is based on the assumption defined by Jien-Wei Yeh and colleagues [7] that the presence of several (five or more) elements in the alloy would increase the configurational entropy of mixing by an amount sufficient to overcome the enthalpies of compound formation, thereby, inhibiting the formation of potentially undesirable additional intermetallics. So, at the increasing of the number of elements in an alloy, the entropic contribution to the total free energy would overcome the enthalpic contribution and, thereby, stabilize solid solution phases [8]. Alloys of systems based on rare earth metals are prone to the formation of solid solutions, which can be both limited and unlimited. In some metallic systems, depending on the composition of the alloys, ordered intermetallic compounds, limited solid solutions, and unlimited solid solutions can be formed simultaneously [9,10,11]. A positive effect of component substitution on electrochemical properties was also observed for Li (Al1–zZnz) solid solution [12].

The concept of stabilizing a crystal structure with high entropy allows us to build electrodes that can be cycled for a long time without significantly reducing the capacity. Previously, this was shown for high-entropy oxides [13] such as cathode materials and MD-HEIP intermetallics as anode materials for Li- and Na-ion batteries [14].

It is shown that increasing entropy by introducing one or more elements leads to a completely different electrochemical behavior and stability of cycles. In addition, there is a unique opportunity to fine-tune the electrochemical characteristics of high-entropy materials, using the different effects of each element on the electrochemical processes. Based on these studies, a possible reaction mechanism is proposed, which takes into account the stabilization of entropy and the maintenance of the invariance of the structure of the initial matrix throughout the cycling process.

In addition to the structural modification of the main phase of the electrode due to the substitution of components [1,2,3,4,5,6,7,8,9,10,11,12,13,14], it is also significant addition of catalysts and CNT conductive additives that improve the cyclic stability of the electrodes. According to known literature data [15,16,17,18,19], the carbon nanotubes (CNTs) are a good candidate material for use in Li-ion batteries due to their perfect electrochemical properties. The insertion of CNTs as a conductive additive at a lower weight loading than conventional carbon black and other graphite representatives to the electrode matrix gives the opportunity to create more quality electrodes.

Chawla et al. [20] described the inclusion of Pd catalyst in CNT cathode materials in Li-O_2_ batteries and received electrochemical data with significant improvement in discharge capacities. The batteries with CNTs and Pd catalyst cycled have 35% more cycles. The activity of the germanium catalyst was investigated to improve the electrochemical performance of SnO_2_/graphene nanocomposite anode material for LIBs [21].

In this study, we implemented two goals: first, we determined the effect of disordering the structure of Y_5−x_Pr_x_Sb_3−y_M_y_ (M = Sn, Pb) electrodes due to component substitution, and second, we determined the effect of a modifying additive (CNT) on the electrochemical activity of electrodes for LIBs and SIBs. A CNT-modified electrode shows better kinetics of Li^+^ /Na^+^ intercalation into electrodes and higher corrosion resistance in electrolyte solutions and, as a result, increases the battery life. The positive effect of these two actions leads to improved cyclic stability and increased electrode capacity up to 30% for LIBs and up to 25% for SIBs.

## 2. Material and Methods

### 2.1. Synthesis and Phase Analysis

The maximally disordered Y_5−x_Pr_x_Sb_3−y_M_y_ (M = Sn, Pb; 0 ≤ x,y ≤ 0.5) alloys were prepared from high purity metals: yttrium (ingot, 99.9 at.%, Sigma-Aldrich, Saint Louis, MO, USA), praseodymium (ingot, 99.9 at.%, Sigma-Aldrich, Saint Louis, MO, USA), antimony (rod, 99.99 at.%, Sigma-Aldrich, Saint Louis, MO, USA), tin (99.8%, shot, 3 mm, Sigma-Aldrich, Saint Louis, MO, USA) and lead (shot, <2 mm, 99.9%, Sigma-Aldrich, Saint Louis, MO, USA). These alloys were synthesized by arc-melting of pure metals under the argon atmosphere. The Y_5−x_Pr_x_Sb_3−y_M_y_(Li/Na)_z_ alloys were synthesized by both thermal and electrochemical methods. For the thermal insertion of lithium and sodium into Y_5−x_Pr_x_Sb_3−y_M_y_, two separate pieces of this alloy were mixed with Li or Na, respectively, according to the aimed stoichiometry of the product and filled into tantalum crucibles under argon atmosphere that were sealed by arc-welding. For the electrochemical method, the lithiation and sodiation of Y_5−x_Pr_x_Sb_3−y_M_y_ alloy were used. The samples synthesized by the thermal method were annealed at 670 K for 24 h. The reaction product was analyzed by X-ray powder diffraction (XRD) using Rigaku MiniFlex 600 powder diffractometer (Cu-radiation), Rigaku, Tokyo, Japan. The phase content of alloys was determined by means of a TESCAN VEGA3 electron microscope (Tescan, Brno, Czech Republic) equipped with Oxford Instruments energy dispersive X-ray analyzer, Aztec ONE system, High Wycombe, UK. In some cases, the flame photometry (Flapho-4 flame photometer, Carl Zeiss Jena, Jena, Germany) for detection of lithium was used.

### 2.2. Structure Refinement

Both the single crystal and powder X-ray diffraction methods were used for the investigation of the crystal structures of Y_5−x_Pr_x_Sb_3−y_M_y_ and Y_5−x_Pr_x_Sb_3−y_M_y_(Li/Na)_z_ phases. Rietveld refinements against X-ray powder diffraction data were performed by using the FULLPROF program (version June 2020) [22]. The single crystals data were collected using an automatic four-circle Xcalibur Oxford Diffraction diffractometer (Agilent Technologies, Inc., Santa Clara, CA, USA) with CCD detector (graphite monochromatized Mo-radiation, ω-scan mode). The analytical absorption corrections were made by CrysalisRed [23]. The crystal structure was solved by direct methods and refined using the SHELX-97 program package (Georg August University of Göttingen, Göttingen, Germany) [24,25].

Crystallographic data (CIF and structure factors) for the structures reported in this paper has been deposited with the Cambridge Crystallographic Data Centre, no. CCDC-2062904 (for Y_5−x_Pr_x_Sb_3−y_M_y_Li_z_) and CCDC-2062905 (for Y_5−x_Pr_x_Sb_3−y_M_y_Na_z_). These data can be obtained free of charge from the Cambridge Crystallographic Data Centre via http://www.ccdc.cam.ac.uk/data_request/cif (accessed on 13 February 2021) [26].

### 2.3. Electrochemical Investigations

Two series, each consisting of five electrodes of the corresponding compositions Y_5_Sb_3_, Y_5−x_Pr_x_Sb_3−y_Sn_y_, Y_5−x_Pr_x_Sb_3−y_Pb_y_, Y_5−x_Pr_x_Sb_3−y_Sn_y_-CNT, and Y_5−x_Pr_x_Sb_3−y_Pb_y_-CNT were prepared for electrochemical studies. Electrodes from Y_5_Sb_3_, Y_5−x_Pr_x_Sb_3−y_Sn_y_, and Y_5−x_Pr_x_Sb_3−y_Pb_y_ were cut from alloys or prepared by grinding of bulk alloys to powders and the following mixed in an agate mortar of 90 wt.% alloys (acting as active material) with 10 wt.% polyvinylidene fluoride (PVDF binder), and finally, these mixtures were pressed onto a stainless-steel grid (about 5 mg, coin cell type electrode 11 mm in diameter and 0.8 mm thick). The electrode with CNT catalyst (Y_5−x_Pr_x_Sb_3−y_Sn_y_-CNT and Y_5−x_Pr_x_Sb_3−y_Pb_y_-CNT) were prepared by mixing powdered alloys with carbon nanotubes in ratio 95:5, 90:10, and 85:15, respectively, and subsequently, sintering at 670 K for 48 h. The carbon nanotubes NC7000 were manufactured by Nanocyl SA (Sambreville, Belgium), which were produced through catalytic chemical vapor deposition (CCVD). Their average diameter and length were 9.5 nm and 1.5 μm, respectively.

Subsequently, the sintering product was ground into a powder to a grain size of almost 1 μm and mixed with 10 wt.% PVDF binder and pressed onto a stainless-steel grid. This grain size of the powder of the active phase of the anode material was due to proportionality with the grain sizes of the powder of the cathode materials, which were commercial.

The lithium-ion cells contain prepared alloy as a working electrode with Li reference and LiCoO_2_ counter electrodes. The sodium-ion cells contain Na as a reference and Na_x_CoO_2_ as counter electrodes, respectively. The ethylencarbonate/dimethylcarbonate nonaqueous electrolyte, containing Li^+^ or Na^+^ ions were used. The anode and cathode were separated by Celgard 2320 separator (Celgrad, Charlotte, CA, USA) impregnated with an electrolyte.

Multi-cycle chronopotentiometry (CP), voltammetry (CVA) and electrochemical impedance spectroscopy (EIS) were performed in three-electrode Swagelok-type cells. All electrochemical measurements were carried out by means of the electrochemical station from CH Instruments (Austin, TX, USA) and 8 Channel Battery Analyzer (BST8-MA, MTI Corporation, Richmond, CA, USA). Galvanostatic charge/discharge cycles were received at *i*_ch_ = 2 mA cm^−2^, *i*_disch_ = 1 mA cm^−2^. The cyclic voltammograms were registered at scan rate = 0.001 V/s. The impedance spectra were taken on electrodes cycled between 1 and 4.5 V using a 0.001 Hz–100 kHz frequency range. To determine the values of the standard deviation for discharge capacity and parameters of EIS measurements, the experiments were repeated three times. The relative standard deviation of discharge capacities does not exceed 8%. The relative standard deviation for all EIS parameters does not exceed 12%.

## 3. Results and Discussion

Disordered and high-entropy alloys are suitable for the development of advanced materials with unique properties that cannot be achieved by conventional materials based only on ordered phases. High-entropy alloys can be used to create materials with high heat resistance and oxidation resistance, with improved mechanical, magnetic, and electrochemical properties. The MD and MD-HEIP were received from Y_5_Sb_3_ by concept based on two mechanisms: *substitution* and *insertion*. For the partial substitution of the Y atoms in crystallographic positions 4*d* and 6*g,* the Pr atoms were used, and Sn or Pb atoms were used for the partial substitution of Sn. As a result of this substitution, a MD phase of Y_5−x_Pr_x_Sb_3−y_M_y_ is formed. The structure of the Y_5_Sb_3_ binary phase contains typical empty octahedral voids in which lithium or sodium can be inserted. Moreover, atoms can be inserted in an octahedral void either thermally or electrochemically.

**Figure 1:**
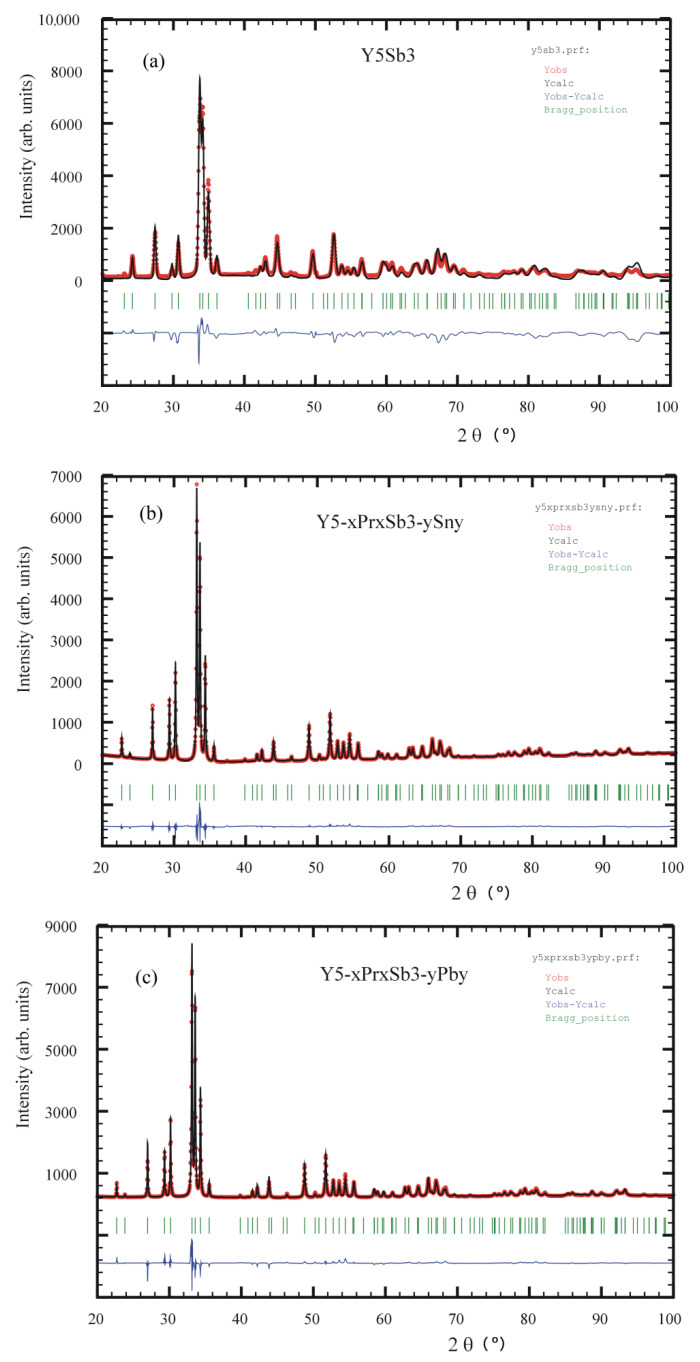
Observed (circles), calculated (line), and difference (**bottom** line) x-ray powder diffraction patterns for of Y_5_Sb_3_ (**a**), Y_5−x_Pr_x_Sb_3−y_Sn_y_ (**b**), and Y_5−x_Pr_x_Sb_3−y_Bi_y_ (**c**) alloys.

**Figure 2:**
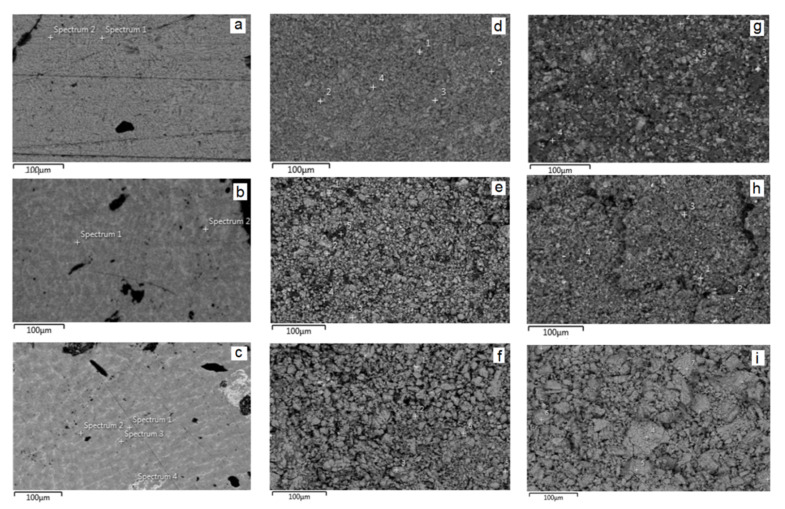
SEM images of Y_5_Sb_3_ (**a**), Y_5−x_Pr_x_Sb_3−y_Sn_y_ (**b**), and Y_5−x_Pr_x_Sb_3−y_Bi_y_ (**c**) alloys and images of these alloys after lithiation (**d**–**f**) and sodiation (**g**–**i**), respectively.

The results of X-ray powder diffraction and microprobe analysis show that the prepared alloys contain the hexagonal phase with Mn_5_Si_3_ structure type [27]. In this structure type, the binary phase Y_5_Sb_3_ crystallizes [28]. The X-ray powder pattern after Rietveld refinements and SEM micrographs of Y_5_Sb_3_, Y_5−x_Pr_x_Sb_3−y_Sn_y_, and Y_5−x_Pr_x_Sb_3−y_Pb_y_ are presented in Figure 1 and Figure 2a–c, respectively. The Rietveld refinements were carried out in *P*6_3_*/mcm* space group with the structure model corresponding to the Mn_5_Si_3_ structure type. The structural data for the ordered Y_5_Sb_3_ and disordered Y_5−x_Pr_x_Sb_3−y_M_y_ (M = Sn, Pb) and experimental details of the structure determination are presented in Table 1. The refined unit cell dimensions of disordered Y_5−x_Pr_x_Sb_3−y_M_y_ phases are increased in comparison to the binary Y_5_Sb_3_ ordered phase; it correlates well with atomic radii of substituting atoms.

For detailed structural analysis and to confirm that the obtained phases were MD-HEIP, a thorough high-precision structural study was performed using single-crystal X-ray methods. Two single crystals were selected from thermally synthesized and homogenized alloys Y_4.5_Pr_0.5_Sb_2.5_Sn_0.5_Li and Y_4.5_Pr_0.5_Sb_2.5_Sn_0.5_Na and used to completely determine the crystal structure. Initial X-ray studies showed that all single crystals had the same hexagonal structure as Hf_5_CuSn_3_-type, which is a filled variant of the Mn_5_Si_3_-type, in which the binary phase of Y_5_Sb_3_ crystallizes. Since these phases are multicomponent, it was important to more accurately determine the distribution of atoms in the crystallographic positions, which allows us to make an X-ray single crystal method. General information on the obtained structural results is summarized in Table 2. Fractional atomic coordinates and displacement parameters for Y_5−x_Pr_x_Sb_3−y_Sn_y_Li and Y_5−x_Pr_x_Sb_3−y_Sn_y_Na are presented in Table 3. Detailed structural refinements show that lithium and sodium can be inserted into octahedral voids.

**Figure 3:**
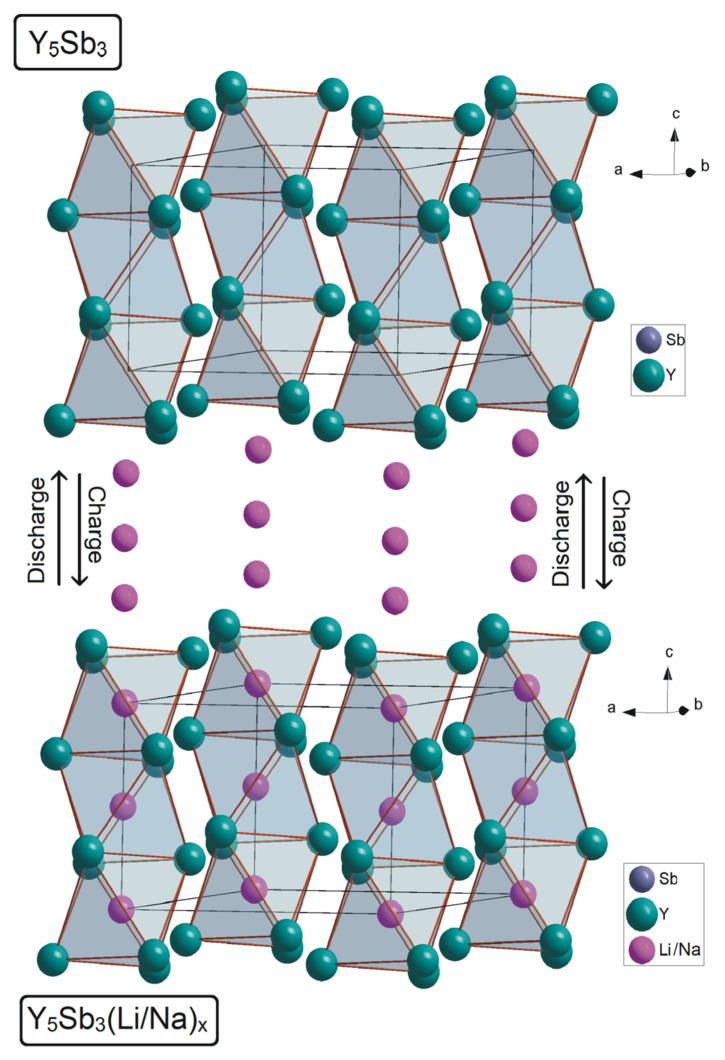
Scheme of insertion of the lithium or sodium atoms into octahedral voids.

The structural feature of these phases based on the possibility of insertion/deinsertion of lithium and sodium atoms into octahedral voids prompted the study of the possibility of carrying out these processes using electrochemical methods. The insertion of lithium or sodium atoms significantly increases the volume of the unit cell, more significantly along the lattice period *c*, since in this direction, the octahedron expands more during the insertion of atoms (Figure 3). At the insertion of Li into octahedra, the volume of the unit cell increases by 4.65 Å^3^, and there are even greater changes up to 11.36 Å^3^ when sodium atoms are inserted.

**Figure 4:**
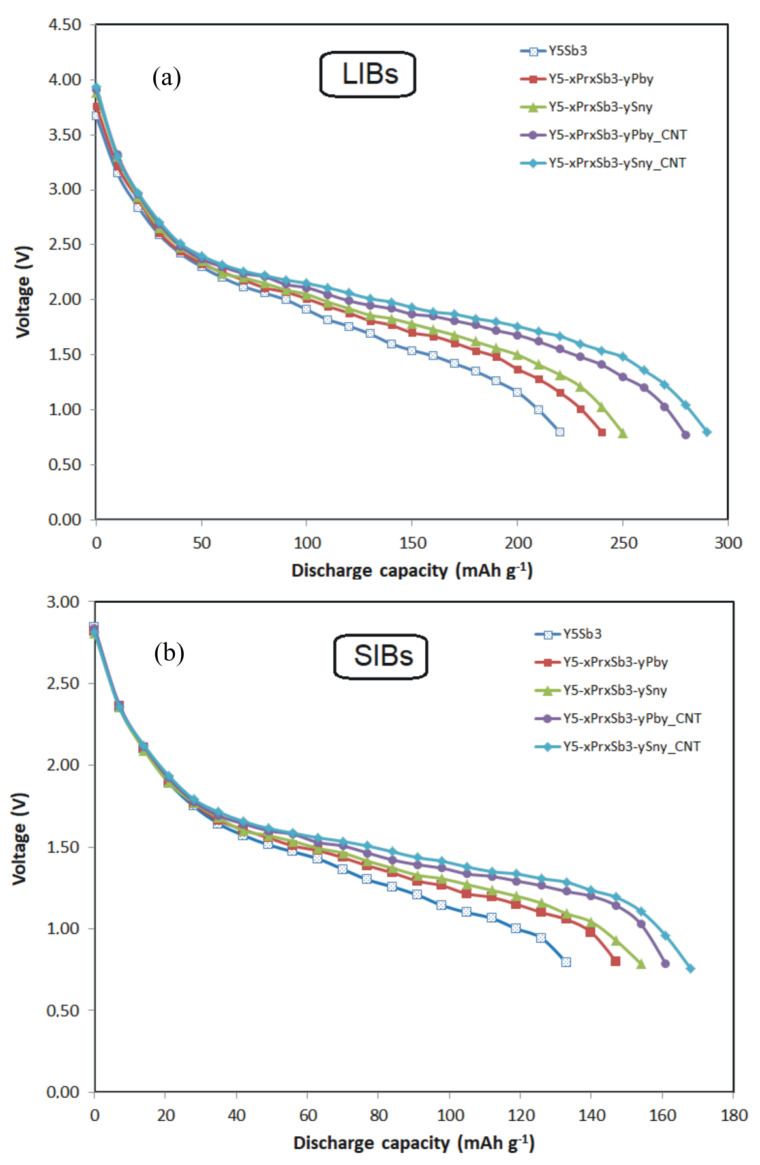
The galvanostatic discharge curve for LIBs (**a**) and SIBs (**b**).

After several initial cycles of electrochemical lithiation/delithiation, as well as sodiation/desodiation of disk electrodes that were cut from the alloy, their amorphization was observed (Figure 2d–i), and their capacity decreased slightly. After 5–8 cycles of activation, the electrodes stabilized and could continue to operate for many cycles without significant loss of capacity. There are no significant differences when using electrodes that were made by pressing powders from crushed alloys. It should be noted that the electrodes from the ordered binary phaseY_5_Sb_3_ have a discharge capacity in the range of 200 mAh · g^−1^ (for LIBs) and 120 mAh · g^−1^ (for SIBs), respectively (Figure 4). The use of electrodes from the MD phases increases the discharge capacitance for both LIBs and SIBs by almost 10%. Moreover, slightly higher values of capacitance have electrodes in which the Sb atoms are replaced by Sn, in comparison with electrodes in which the Sb are replaced by Pb atoms.

**Figure 5:**
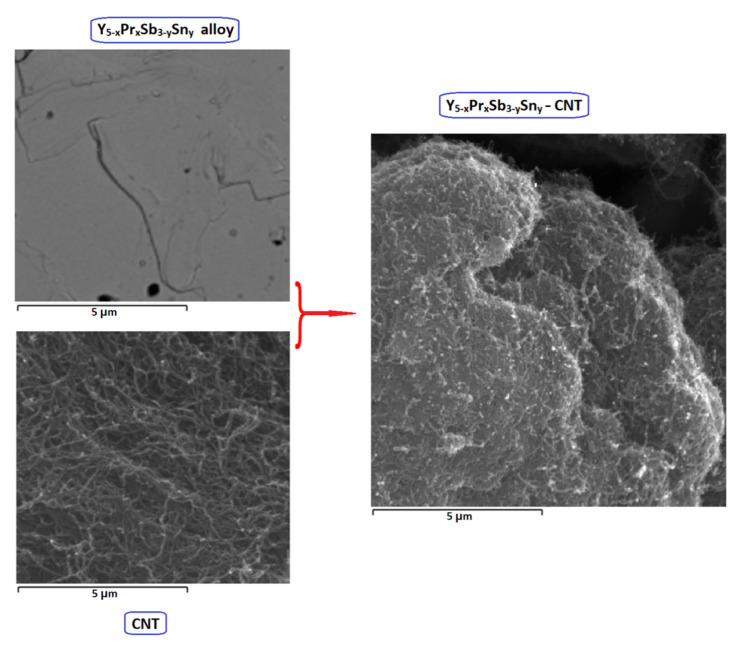
SEM images of initial Y_5-x_Pr_x_Sb_3-y_Sn_y_ alloy, CNT, and modified Y_5−x_Pr_x_Sb_3−y_Sn_y_–CNT after sintering.

The next important step was the modification of the electrodes by carbon nanotubes in order to increase their capacity and improve cyclic stability. This modification was performed for MD phases of Y_5−x_Pr_x_Sb_3−y_Sn_y_ and Y_5−x_Pr_x_Sb_3−y_Pb_y_. Different amounts of CNT were added, namely 5, 10, and 15 wt.% to alloys with subsequent mixing, pressing, and sintering. For example, Figure 5 shows the SEM images of the initial alloy, nanotubes, and the product of their sintering. It is seen that the nanotubes wrap the grains of the alloy, filling the intergranular space.

**Figure 6:**
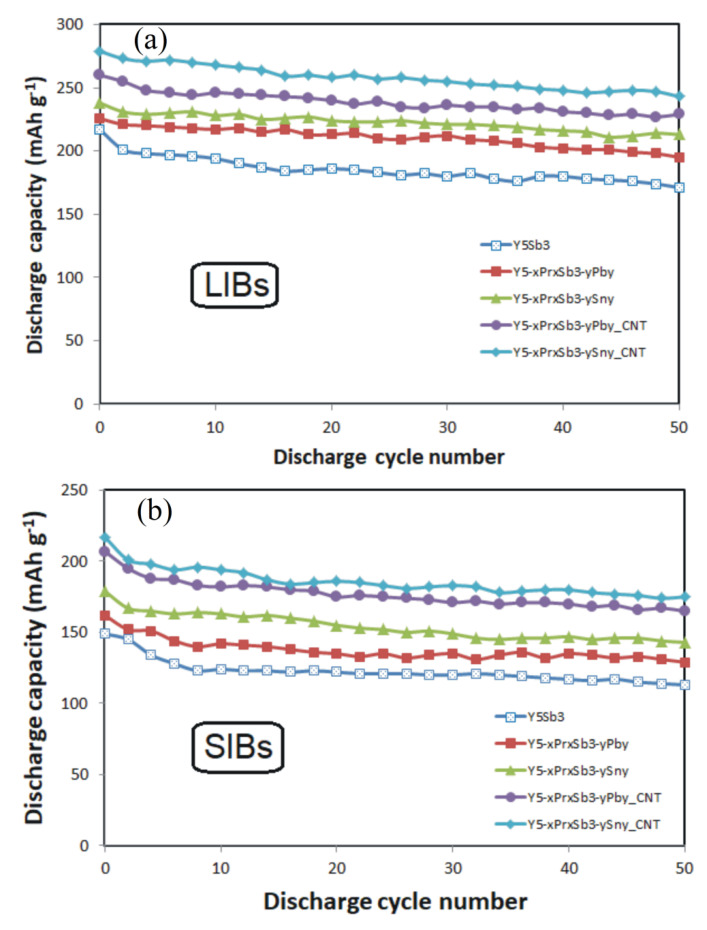
Specific capacities vs. cycle number of the tested alloys for LIBs (**a**) and SIBs (**b**).

Electrochemical studies have shown that the optimal amount of CNT is 10 wt.%. It has been experimentally established that if the content of carbon nanotubes in the electrodes exceeds 10 wt.%, for example, 15 wt.%, the capacity and cyclic characteristics of the electrodes begin to deteriorate due to the formation of grains of carbide phases during sintering. It is also important to ensure the mechanical stability of the electrodes so that they do not crack during numerous charge–discharge cycles. This is realized by adding a binder material, the optimal amount of which was 10 wt.%. The addition of CNTs and PVDF binder to electrodes improved capacity and their cycles’ stability. The CNTs provided diffusion pathways for Li+ or Na+ ions to most of the grains inside the material, improving charge/discharge kinetics, and the PVDF binder provided good adhesion between the electrode materials and current collectors, giving excellent electrochemical, mechanical, and thermal stability of electrodes. Modification of alloys with carbon nanotubes allowed us to significantly increase the discharge capacity of both types of batteries as LIBs and SIBs (Figure 4). Modification of Y_5−x_Pr_x_Sb_3−y_M_y_ (M = Sn, Pb) alloys by carbon nanotubes allowed us to significantly increase the discharge capacity of both types of batteries, which reached 280 mAh · g^−1^ (for LIBs) and 160 mAh · g^−1^ (for SIBs), respectively. These maximum capacities are for Y_5-x_Pr_x_Sb_3-y_Sn_y_ alloy electrodes in which antimony is partially replaced by tin. For Y_5-x_Pr_x_Sb_3-y_Pb_y_ alloys in which antimony is replaced by lead, these capacities are slightly smaller and are 270 mAh · g^−1^ (for LIBs) and 155 mAh · g^−1^ (for SIBs), respectively. The study of cyclic stability shows that for CNT-modified electrodes after 50 cycles of charge–discharge, capacity decreases by no more than 8% (Figure 6).

**Figure 7:**
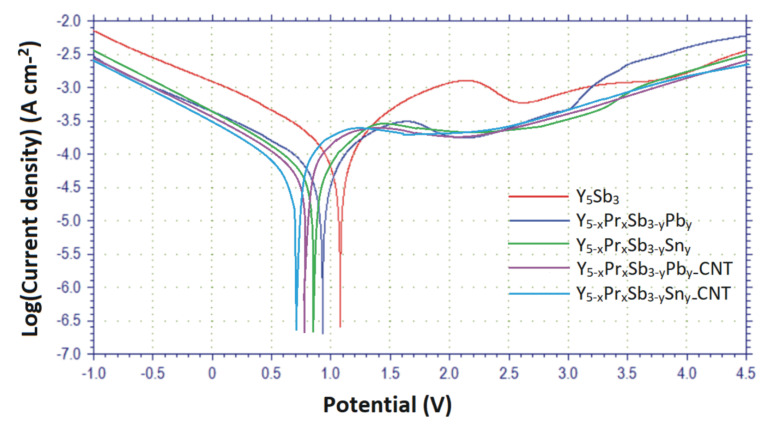
Potentiokinetic polarization curves of the tested alloys for LIBs.

The analysis potentiodynamic polarization curves (Figure 7) show that tested Y_5_Sb_3_, Y_5−x_Pr_x_Sb_3−y_M_y_ (M = Sn, Pb), and Y_5−x_Pr_x_Sb_3−y_M_y_ (M = Sn, Pb)–CNT electrodes passivate in LiPF_6_ based ethylencarbonate/dimethylcarbonate electrolyte, electrolyte solution, and their passivation range extends from +1.07 V to +0.70 V. The electrode corrosion potential (*E*_corr_) is equal to +1.07 V for Y_5_Sb_3_ and shifts to +0.97 V for Y_5−x_Pr_x_Sb_3−y_Pb_y_ and +0.84 V for Y_5−x_Pr_x_Sb_3−y_Sn_y_. For the catalyst activated by CNT, Y_5−x_Pr_x_Sb_3−y_Pb_y_–CNT and Y_5−x_Pr_x_Sb_3−y_Sn_y_–CNT electrodes, the *E*_corr_ shifts to +0.77 V and +0.70 V, respectively, and these shifts indicate an increase of corrosion resistance in the electrolyte solution, which causes a higher specific capacity and better cyclic stability.

**Figure 8:**
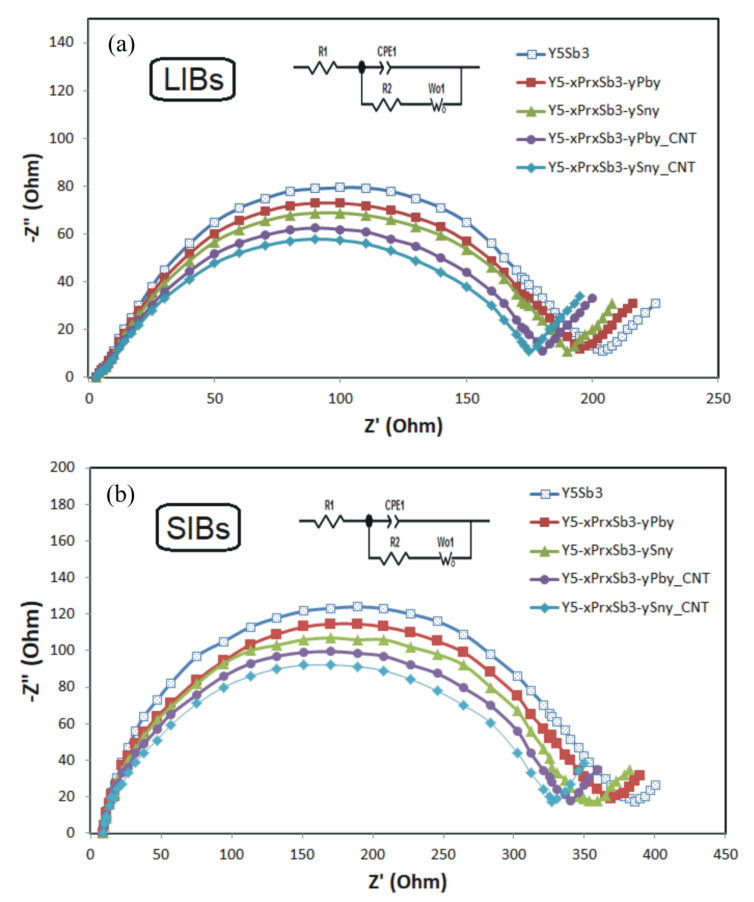
Nyquist plot from electrochemical impedance spectroscopy (EIS) data of the tested alloys for LIBs (**a**) and SIBs (**b**). EIS data analyzed by fitting to an equivalent electrical circuit scheme (inset) in which R1 is the electrolyte solution resistance, R2 is the charge transfer resistance, CPE1 is the double-layer capacitance, and Wo1 is related to the diffusion-controlled Warburg impedance.

Figure 8 shows the Nyquist plot from electrochemical impedance spectroscopy (EIS) data of Y_5_Sb_3_, Y_5-x_Pr_x_Sb_3-y_M_y_ (M = Sn, Pb), and Y_5−x_Pr_x_Sb_3−y_M_y_–CNT (M = Sn, Pb) electrodes for both LIBs and SIBs. The semi-circle areas correspond to the charge transfer limitation. Generally, the kinetics of Li+ ions intercalation into electrodes is faster than Na+ because of the smaller radius and lighter mass of Li+ compared to Na+, resulting in smaller charge transfer resistances in the LIBs (Figure 8a) than in the SIBs (Figure 8b). As shown in Figure 8 for LIBs, the semi-circle areas of Y_5−x_Pr_x_Sb_3−y_M_y_–CNT (M = Sn, Pb) electrodes are smaller than those of the Y_5_Sb_3_ and Y_5−x_Pr_x_Sb_3−y_M_y_, which demonstrates that the charge-transfer reaction is facilitated for the modified electrode. Similar behavior of these electrodes is observed for SIBs, but the higher values of the resistances indicate that the processes of charge transfer in this system are more difficult.

The electrochemical lithiation and sodiation of Y_5−x_Pr_x_Sb_3−y_M_y_ phase can be written, respectively, as:

Y_5−x_Pr_x_Sb_3−y_M_y_ + *z*Li^+^ + *z*e^-^ ↔ [Y_5−x_Pr_x_Sb_3−y_M_y_…*z*Li] ↔ Y_5−x_Pr_x_Sb_3−y_M_y_Li_z_ (I)

Y_5−x_Pr_x_Sb_3−y_M_y_ + *z*Na^+^ + *z*e^-^ ↔ [Y_5−x_Pr_x_Sb_3−y_M_y_…*z*Na] ↔ Y_5−x_Pr_x_Sb_3−y_M_y_Li_z_ (II)

CNT additives fill the intergranular space during sintering and improve the electrical connectivity within the electrode, thus, improving capacity by connecting and maintaining more electron conduction pathways between the active material and the current collector and then, finally, increasing the specific capacities and cycle stability of electrodes.

## 4. Conclusions

The maximally disordered Y_5−x_Pr_x_Sb_3−y_M_y_ (M = Sn, Pb) phases with stable hexagonal Mn_5_Si_3_ structure type were prepared and investigated as the anode materials for LIBs and SIBs. During the electrochemical lithiation and sodiation, the formation of Y_5−x_Pr_x_Sb_3−y_M_y_Li_z_ and Y_5−x_Pr_x_Sb_3−y_M_y_Na_z_ maximally disordered–high entropy intermetallic phases, as the result of the insertion of Li/Na into octahedral voids, were observed. Carbon nanotubes are an effective additive to improve the cycle stability of the Y_5−x_Pr_x_Sb_3−y_M_y_ (M= Sn, Pb) anodes for lithium-ion and sodium-ion batteries. Modification of Y_5−x_Pr_x_Sb_3−y_M_y_ (M = Sn, Pb) alloys by structure disordering and carbon nanotube additives allowed us to significantly increase the discharge capacity of both types of batteries, which reached 280 mAh · g^−1^ (for LIBs) and 160 mAh · g^−1^ (for SIBs), respectively. A CNT-modified electrode also shows higher corrosion resistance in electrolyte solutions and, as a result, demonstrates an increased battery life. The effect of the addition of CNTs and PVDF binder on the improvement of capacity and cycle stability of electrodes is due to the following factors: CNTs provide diffusion pathways for Li+ or Na+ ions to most of the grains inside the material, improving charge/discharge kinetics; PVDF provides good adhesion between the electrode materials and current collectors, giving excellent electrochemical, mechanical, and thermal stability of electrodes.

## Figures and Tables

**Table 1:** Crystallographic data for the Y_5_Sb_3_ and Y_5-x_Pr_x_Sb_3-y_M_y_ (M = Sn, Pb) and experimental details of the structure determination.

Phase		2. Y_5_Sb_3_	3. Y_5−x_Pr_x_Sb_3−y_Sn_y_	4. Y_5−x_Pr_x_Sb_3−y_Pb_y_
5. Composition (at.%) from XRD		6. Y_62.5_Sb_37.5_	7. Y_57.1_Pr_5.4_Sb_32.2_Sn_5.3_	8. Y_57.0_Pr_5.5_Sb_32.4_Pb_5.1_
9. Composition (at.%) from EPMA		10. Y_62.3_Sb_37.7_	11. Y_56.8_Pr_5.7_Sb_31.9_Sn_5.6_	12. Y_57.2_Pr_5.3_Sb_32.5_Pb_5.0_
13. Diffractometer; radiation		14. HZG-4a, Cu Kα	15. HZG-4a, Cu Kα	16. HZG-4a, Cu Kα
17. 2θ range, deg		18. 20÷100	19. 20÷100	20. 20 ÷ 100
21. Step size, deg.; counting time, s.		22. 0.03, 20	23. 0.03, 20	24. 0.03, 20
25. Structure type		26. alic>26. Mn_5_Si_3__.5_	27. alic>27. Mn_5_Si_3_	28. alic>28. Mn_5_Si_3_
29. Space group		30. *P*6_3_/*mcm*	31. *P*6_3_/*mcm*	32. *P*6_3_/*mcm*
33. Pearson symbol		34. hP16	35. hP16	36. hP16
37. Unit cell dimensions:		38.		39.
40. *a*, Å		41. 8.8892(2)	42. 9.0325(1)	43. 9.0418(2)
44. *c*, Å		45. 6.3256(1)	46. 6.5958(1)	47. 6.6023(1)
48. alic>48. *V*, Å^3^		49. 432.87(2)	50. 466.03(1)	51. 467.45(2)
52. Reliability factors:		53.		54.
55. R_F_ (%); R_B_ (%)		56. 6.02, 7.83	57. 5.17, 6.06	58. 5.33, 7.28
59. R_P_ (%)		60. 4.92	61. 3.17	62. 3.23
63. R_wp_ (%)		64. 8.19	65. 4.65	66. 5.30
67. ^χ2^		68. 2.13	69. 1.07	70. 0.97
71. Atomic parameters (*xyz*):	72. 4*d* 73. 6*g* 74. 6*g*	75. Y1 1/3 2/3 0 76. Y2 0.2363(1) 0 1/4 77. Sb 0.5997(2) 0 1/4	78. (Y/Pr)1 1/3 2/3 0 79. (Y/Pr)2 0.2406(1) 0 1/4 80. (Sb/Sn) 0.6036(2) 0 1/4	81. (Y/Pr)1 1/3 2/3 0 82. (Y/Pr)2 0.2438(1) 0 1/4 83. (Sb/Pb) 0.6062(2) 0 1/4

**Table 2:** Crystallographic data for Y_5-x_Pr_x_Sb_3-y_Sn_y_Li and Y_5-x_Pr_x_Sb_3-y_Sn_y_Na single crystals and experimental details of the structure determination.

84. Phase	85. Y_5−x_Pr_x_Sb_3−y_Sn_y_Li	86. Y_5−x_Pr_x_Sb_3−y_Sn_y_Na
87. Formula	88. Y_4.5_Pr_0.5_Sb_2.5_Sn_0.5_Li	89. Y_4.5_Pr_0.5_Sb_2.5_Sn_0.5_Na
90. Structure type	91. Hf_5_CuSn_3_	92. Hf_5_CuSn_3_
93. Formula weight (g/mol)	94. 841.22	95. 857.27
96. Space group	97. *P*6_3_/*mcm*	98. *P6_3_*/*mcm*
99. Pearson symbol	100. hP18	101. hP18
102. Crystal dimensions (mm^3^)	103. 0.04 × 0.05 × 0.09	104. 0.03 × 0.04 × 0.08
105. Unit cell dimensions:	106.	107.
108. *a*, Å	109. 9.0614(1)	110. 9.0978(1)
111. *c*, Å	112. 6.6192(1)	113. 6.6599(1)
114. *V*, Å^3^	115. 470.68(1)	116. 477.39(1)
117. Calculated density (D_calc,_ g·sm^−3^)	118. 5.935	119. 5.964
120. Absorption coefficient (µ, mm^−1^)	121. 38.203	122. 37.714
123. Scan mode	124. Ω	125. ω
126. Theta range for data collection (deg.)	127. 2.60–26.69	128. 2.59–26.31
129. *F*(000)	130. 721	131. 737
132. Range in *h k l*	133. −11 ≤ *h* ≤ 11,134. −11 ≤ *k* ≤ 11,135. −7 ≤ *l* ≤ 7	136. −11 ≤ *h* ≤ 11,137. −11 ≤ *k* ≤ 11,138. −7 ≤ *l* ≤ 7
139. Total no. reflections	140. 1544	141. 1522
142. Reflections with *I* > 2σ(*I*)	143. 190 (*R*_sigma_ = 0.0114)	144. 189(*R*_sigma_ = 0.0180)
145. Data/parameters	146. 190/12	147. 189/13
148. Goodness-of-fit on *F^2^*	149. 1.144	150. 1.169
151. Final *R* indices [*I* > 2σ(*I*)]	152. *R*_1_ = 0.0341	153. *R*_1_ = 0.0476
154.	w*R*_2_ = 0.0936	155. w*R*_2_ = 0.1296
156. Largest diff. peak/hole (e/Å^3^)	157. 2.204/−1.901	158. 2.622/−1.981

**Table 3:** Fractional atomic coordinates and displacement parameters for Y_5−x_Pr_x_Sb_3−y_Sn_y_Li and Y_5−x_Pr_x_Sb_3−y_Sn_y_Na (Å^2^).

159. Atom	160. Site	161. x/a	162. y/b	163. z/c	164. *U*_iso_ */*U*_eq_	165. Occ. (<1)
166. Y_5−x_Pr_x_Sb_3−y_Sn_y_Li						
167. Y1	168. 6*g*	169. 0.2696(2)	170. 0.2696(2)	171. 1/4	172. 0.0233(6)	173. 0.80
174. Pr1	175. 6*g*	176. 0.2696(2)	177. 0.2696(2)	178. 1/4	179. 0.0233(6)	180. 0.20
181. Y2	182. 4*d*	183. 2/3	184. 1/3	185. 0	186. 0.0180(6)	187. 0.88
188. Pr2	189. 4*d*	190. 2/3	191. 1/3	192. 0	193. 0.0180(6)	194. 0.12
195. Sb1	196. 6*g*	197. 0.63128(12)	198. 0.63128(12)	199. 1/4	200. 0.0084(5)	201. 0.80
202. Sn1	203. 6*g*	204. 0.63128(12)	205. 0.63128(12)	206. 1/4	207. 0.0084(5)	208. 0.20
209. Li1	210. 2*b*	211. 0	212. 0	213. 0	214. 0.002 *	215. 1.00
216.	*U* ^11^	217. *U*^22^	218. *U*^33^	219. *U*^12^	220. *U*^13^	221. *U*^23^
222. Y1	223. 0.0224(7)	224. 0.0224(7)	225. 0.0256(11)	226. 0.0116(8)	227. 0	228. 0
229. Pr1	230. 0.0224(7)	231. 0.0224(7)	232. 0.0256(11)	233. 0.0116(8)	234. 0	235. 0
236. Y2	237. 0.0171(7)	238. 0.0171(7)	239. 0.0196(13)	240. 0.0086(3)	241. 0	242. 0
243. Pr2	244. 0.0171(7)	245. 0.0171(7)	246. 0.0196(13)	247. 0.0086(3)	248. 0	249. 0
250. Sb1	251. 0.0078(5)	252. 0.0078(5)	253. 0.0110(7)	254. 0.0049(5)	255. 0	256. 0
257. Sn1	258. 0.0078(5)	259. 0.0078(5)	260. 0.0110(7)	261. 0.0049(5)	262. 0	263. 0
264. Y_5−x_Pr_x_Sb_3−y_Sn_y_Na						
265. Y1	266. 6*g*	267. 0.2704(3)	268. 0.2704(3)	269. 1/4	270. 0.0261(9)	271. 0.80
272. Pr1	273. 6*g*	274. 0.2704(3)	275. 0.2704(3)	276. 1/4	277. 0.0261(9)	278. 0.20
279. Y2	280. 4*d*	281. 2/3	282. 1/3	283. 0	284. 0.0261(10)	285. 0.76
286. Pr2	287. 4*d*	288. 2/3	289. 1/3	290. 0	291. 0.0261(10)	292. 0.24
293. Sb1	294. 6*g*	295. 0.63151(18)	296. 0.63151(18)	297. 1/4	298. 0.0113(8)	299. 0.80
300. Sn1	301. 6*g*	302. 0.63151(18)	303. 0.63151(18)	304. 1/4	305. 0.0113(8)	306. 0.20
307. Na1	308. 2*b*	309. 0	310. 0	311. 0	312. 0.008 *	313. 0.98
314.	*U* ^11^	315. *U*^22^	316. *U*^33^	317. *U*^12^	318. *U*^13^	319. *U*^23^
320. Y1	321. 0.0267(12)	322. 0.0267(12)	323. 0.0280(16)	324. 0.0152(12)	325. 0	326. 0
327. Pr1	328. 0.0267(12)	329. 0.0267(12)	330. 0.0280(16)	331. 0.0152(12)	332. 0	333. 0
334. Y2	335. 0.0241(11)	336. 0.0241(11)	337. 0.031(2)	338. 0.0121(6)	339. 0	340. 0
341. Pr2	342. 0.0241(11)	343. 0.0241(11)	344. 0.031(2)	345. 0.0121(6)	346. 0	347. 0
348. Sb1	349. 0.0102(9)	350. 0.0102(9)	351. 0.0140(11)	352. 0.0053(7)	353. 0	354. 0
355. PB1	356. 0.0102(9)	357. 0.0102(9)	358. 0.0140(11)	359. 0.0053(7)	360. 0	361. 0

U_eq_ is defined as one third of the trace of the orthogonalized U_ij_ tensor. The anisotropic displacement factor exponent takes the form: *Uij* = −2*π^2^*[(h^2^a*)*^2^U*_11_ *+ ...+* 2hka*b*U_12_]. *U*_13_ = *U*_23_ = 0.

## Data Availability

Data is contained within the article.
